# Reserve-building as a buffer for depression among individuals living with disability: a longitudinal study of current activities related to brain health

**DOI:** 10.3389/fpsyg.2024.1330437

**Published:** 2024-02-22

**Authors:** Carolyn E. Schwartz, Katrina Borowiec, Bruce D. Rapkin

**Affiliations:** ^1^DeltaQuest Foundation, Inc., Concord, MA, United States; ^2^Departments of Medicine and Orthopaedic Surgery, Tufts University Medical School, Boston, MA, United States; ^3^Department of Measurement, Evaluation, Statistics, and Assessment, Boston College Lynch School of Education and Human Development, Chestnut Hill, MA, United States; ^4^Department of Epidemiology and Population Health, Albert Einstein College of Medicine, Bronx, NY, United States

**Keywords:** depression, employment, reserve-building, disability, COVID, protective factors

## Abstract

**Aims:**

This study examined whether reserve-building activities are associated with attenuated reported depression among people who were disabled from work due to a medical condition as compared to employed, retired, and unemployed participants.

**Methods:**

This secondary analysis included 771 individuals who provided data at three time points: baseline (late Spring 2020), follow-up 1 (Spring 2021), and follow-up 2 (Fall 2021). The DeltaQuest Reserve-Building Measure assessed current activities related to brain health. An analysis of variance and Pearson correlation coefficients assessed group differences in reserve-building activity scores. Classification and regression tree (CART) modeling investigated factors associated with higher and lower reported depression by employment group. The random effects (RE) models tested two buffering hypotheses: (1) comparing all groups to the employed group and (2) examining within-group effects.

**Results:**

Engaging in outdoor activities, exercise, and religious/spiritual activities was associated with reduced depression over time in the overall sample. While disabled participants endorsed lower levels of being Active in the World, Outdoor activities, and Exercise and higher levels of Inner Life and Passive Media Consumption than the other employment groups, more reserve-building activities distinguished depression levels in the disabled group's CART models compared to the others. Among the disabled, unemployed, and retired participants, engaging in any reserve-building activities was also associated with lower depression scores, which was distinct from the employed participants. In the RE models that used the employed group as the reference category, only the disabled group's level of depression was buffered by engaging in creative activities. In the within-group RE models, the disabled group's engagement in Religious/Spiritual, Outdoors, and Games was associated with substantially reduced within-group depression, which was different from the other employment groups. In contrast, reserve-building activities were not implicated at all as buffers for employed participants.

**Conclusion:**

This study revealed a beneficial effect of reserve-building activities on buffering depression over time during the COVID-19 pandemic, particularly for disabled people. It documented that even if such individuals engaged in lesser amounts of such activities as compared to other employment groups, the buffering effect was substantial. Given the low-cost and accessible nature of reserve-building activities, it would be worthwhile to encourage such activities for disabled individuals.

## Introduction

There is a substantial and growing body of evidence suggesting that engaging in cognitively stimulating activities enhances one's resilience to morbidity and even to the long-term effects of disabling conditions (Brochet, 2018). Early research on “cognitive reserve” focused on educational attainment as a proxy for premorbid intelligence (Stern et al., 1994). Over time, both understanding and measurement of targeted “leisure activities” (Stern, 2009) or “reserve-building activities” (Schwartz et al., 2018a) grew more sophisticated. These activities comprised cognitive, physical, social, and even spiritual activities, all of which were posited to stimulate different parts of the brain (Schwartz et al., 2016b). Research demonstrated the beneficial effects of reserve-building on health outcomes in traumatic brain injury (Nunnari et al., 2014; Mathias and Wheaton, 2015), Alzheimer's disease (Stern et al., 1994), Parkinson's disease (Hindle et al., 2014), multiple sclerosis (Sumowski et al., 2009a; Schwartz et al., 2016a), HIV-related dementias (Cody and Vance, 2016), stroke (Nunnari et al., 2014), spinal disorders (Schwartz et al., 2021), heterogeneous chronic-illness samples (Schwartz et al., 2018b, 2019), and normal aging (Lövdén et al., 2005, 2012). These effects were revealed by self-reported data on such activities (Schwartz et al., 2013a,b) and by magnetic resonance imaging studies (Lövdén et al., 2005; Sumowski et al., 2009b, 2010; Schwartz et al., 2016a).

While reserve-building research has focused on how such activities can improve health outcomes, in general, and cognitive function, in particular, among neurological cohorts where cognitive disability is a great concern (Brochet, 2018), recent research has also documented the connection between reserve and depression. A recent meta-analysis of predominantly cross-sectional studies revealed that engaging in reserve-building activities is associated with reduced depressive symptoms among older adults (Kim and Park, 2021). Furthermore, the reserve-cognitive function connection is mediated by depression in people with multiple sclerosis (Patel et al., 2018). Depression causes significant changes in behavior, which can reduce one's engagement in reserve-building pursuits. Treating depression can thus be important for enabling a more active lifestyle and thereby offsetting the cognitive burden of the disease (Patel et al., 2018). Engaging in healthy lifestyle behaviors, including reserve-building physical exercise, is associated with less severe coronavirus disease 2019 (COVID-19) and a shorter recovery duration (Yagmaee et al., 2023).

One wonders, however, whether engaging in reserve-building pursuits can serve as a buffer against depression. Depression is one of the leading causes of disease worldwide (Richards, 2011) and the fourth leading cause of disease burden worldwide (Üstün et al., 2004). It affects 8% of individuals in the United States (Brody et al., 2018) and 12% of individuals in Europe (Copeland et al., 2004) and accounts for 4.4% of total disability-adjusted life years worldwide (Üstün et al., 2004). It affects a substantial proportion of caregivers of individuals with disabilities (Ebrahimi et al., 2021). Although pharmacologic treatment of depression is available, the effectiveness of such treatments varies across individuals and depression subtypes (Gabriel et al., 2020), and treatment side effects are notable (Read et al., 2014; Read and Williams, 2018). For example, serotonin selective uptake inhibitors may not only blunt the negative effect but also dampen positive emotions, which results in people with depression not “feeling like themselves” (Goodwin et al., 2017). Finding non-pharmacologic alternatives for treating depression would be beneficial and empowering.

While there are many reasons for people to experience periods of depression, the recent COVID-19 pandemic may be considered a powerful cause. Depression symptom prevalence was threefold higher during the COVID-19 pandemic as compared to a previous study (Ettman et al., 2020), with a higher risk of depressive symptoms associated with lower income, having less money in savings, and exposure to more stressors (Ettman et al., 2020). Many factors may play a role in this rising prevalence, including grief over the loss of loved ones (Eisma et al., 2021), social isolation (Pietrabissa and Simpson, 2020), and long-term symptoms of COVID-19 (Penninx, 2021), among others. In earlier research done by our group and on which the present study builds, we found that employment status was an important predictor of depression (Schwartz et al., 2023a,b). Specifically, people who were disabled from work due to a medical condition were more likely to be depressed throughout the pandemic than other employment groups (Schwartz et al., 2023a,b). Even after adjusting the longitudinal models for COVID-19-specific stressors (e.g., hardship, worry, and low social support) and cognitive appraisal processes, the association between being disabled and being depressed remained highly significant (Schwartz et al., 2023b). This lack of mediation suggests that other unmeasured factors are contributing to their reported depression.

The present study thus aimed to examine whether a noted salutogenic factor—reserve-building activities—might play a role in attenuating reported depression among disabled participants. The study addressed three research questions:

1. What reserve-building activities differentiate levels of baseline depression during the COVID-19 pandemic for people who are employed, unemployed, retired, or disabled from work due to a medical condition?
2. Do reserve-building activities buffer depression trajectories during the COVID-19 pandemic for people who are unemployed, retired, or disabled as compared to those who are employed? In other words, relative to employed people, are there group differences in the relationship between baseline reserve-building activities and depression over time?
3. Do reserve-building activities buffer depression trajectories during the COVID-19 pandemic within employment groups? In other words, relative to an individual's personal depression mean, how does their baseline reserve-building predict variation or deviation around their average depression over time?

## Methods

### Sample and design

This secondary analysis utilized data collected for a longitudinal study of the psychosocial impact of the COVID-19 pandemic (see Ref. Schwartz et al., 2023a for details). The study sample included 771 individuals who provided data at three time points: baseline (late Spring 2020), follow-up 1 (Spring 2021), and follow-up 2 (Fall 2021). Table 1 provides the sociodemographic characteristics of the study sample.

Table 1Demographic characteristics of study participants at baseline (*n* = 771).

**Table:** 

**Variable**	**%**
Gender: % female	82
Marital status	
Never married	15
Married/cohabiting	63
Separated/divorced	16
Widowed	6
Hispanic ethnicity	1
White race	94
Had COVID	5
Education	
High school diploma or less	7
Trade or technical school	6
Some college	23
Bachelors degree	34
Graduate or professional degree	29
Employment status at baseline	
Currently working	36
Retired	30
Unemployed	11
Disabled due to medical condition	23
Difficulty paying bills	
Not at all	59
Slightly	22
Moderately	10
Very	6
Extremely	3

**Table:** 

	**Mean**	**SD**
Age	55.56	13.45
No. of comorbid conditions^*^	3.31	2.13
Years since diagnosis	15.28	13.64
Body Mass Index	29.57	8.53

^*^Excluding depression.

### Measures

*Depression* was measured by a depression index created using items from existing measures that reflected similar content to the Patient Health Questionnaire-8 (Kroenke et al., 2009). A score of 50 or higher reflected being significantly depressed on this index. See Ref. Schwartz et al. (2023a) for full details.

*Reserve-Building Activities* were assessed with the DeltaQuest Reserve-Building Activities measure (Schwartz et al., 2018a) using the indicators of current reserve-building activities subscales. These subscales comprise activities posited to build reserves and activities that take up discretionary time but are not posited to build reserves. The reserve-building subscales are Active in the World (e.g., attending lectures; three items), Games (e.g., puzzles; three items), Outdoors (e.g., spending time outdoors; three items), Creative (e.g., hobbies involving working with one's hands; four items), Religious/Spiritual (e.g., individual or group religious activities; three items), Exercise (e.g., mild, moderate, and strenuous exercise; four items), Inner Life (e.g., reading; three items), and Shopping/Cooking (e.g., prepared food as a hobby; two items). The Passive Media Consumption subscale assesses ineffective activities that are not posited to influence reserve (e.g., watching television; three items) and is included in the measure because time allocated to passive media would take away time from effective reserve-building pursuits. The measure has documented reliability and validity (Schwartz et al., 2018a) and has been used in other studies and samples evaluating the link between reserve-building, health, and social determinants of health (Schwartz et al., 2018b, 2019).

*Demographic characteristics* included age, gender, years since diagnosis, race, ethnicity, education, difficulty paying bills, employment status, cohabitation/marital status, height and weight (to compute body mass index), and comorbidities (excluding depression).

### Statistical analysis

Descriptive statistics summarized the demographic characteristics of the sample, and analysis of variance (ANOVA) investigated raw employment-group differences in reserve-building activities. Pearson correlation coefficients were used to evaluate whether multicollinearity might be an issue for the nine reserve-building subscales if they were included in multivariate models [i.e., if *r* ≥ 0.80 (Berry and Feldman, 1985)], as well as notable patterns in the intercorrelations in the overall sample and by employment group. Three sets of analyses were then done to investigate each of the three research questions.

#### Reserve-building activities that differentiate subgroup-specific levels of baseline depression

Classification and regression tree (CART) modeling was used to investigate employment-group differences in depression related to current reserve-building activities (Research Question #1). CART analysis is a non-parametric decision tree methodology that segments the study sample into meaningful and homogeneous subgroups (Lemon et al., 2003). Our use of CART was aimed at interaction identification, that is, to identify relationships that pertain only to specific subgroups, and specify these relationships in a formal parametric model (IBM, 2021).

We tested CART models predicting the depression index, which were computed separately by employment group at baseline. These models used as independent variables current reserve-building activities related to positive vs. ineffective ways of spending discretionary time (Active in the World, Games, Outdoors, Exercise, Creative, Religious and Spiritual, Inner Life, and Shopping and Cooking vs. Passive Media Consumption).

In growing the trees, we selected a minimum parent size of 40, a maximum child size of 20, a maximum tree depth of 5, and a CART minimum for improvement of 0.0001. This is the minimum decrease in impurity required to split a node, and the SPSS default is 0.0001. Higher values tend to produce trees with fewer nodes. CART analyses were implemented on baseline data.

#### Reserve-building activities as a buffer for depression trajectories over time

Random effects (RE) models (Laird and Ware, 1982) examined the effects of employment status and its intersection with RB activities on depression scores over the 15.5 months of follow-up. These models were computed as a layered series as follows using the MIXED procedure in SPSS. The models included fixed slopes. Intercepts had both a fixed and a random component. The dependent variable of the RE models was the continuous depression index. The null model included the intercept and time from baseline. Additional variables were added in the following sequence. Unless otherwise stated (i.e., “at baseline”), the independent variables were time-varying covariates.

RE Model 1 (Null Model): time from baseline.
RE Model 2: demographic covariates were added, and the significant demographic covariates were retained for subsequent models.
RE Model 3: COVID-19 infection status at baseline was added.
RE Model Series 4: reserve-building variables at baseline were added as main effects.
RE Model Series 5: two-way interactions of employment groups by reserve-building variables at baseline.

To address the second research question, the RE models included the whole sample, and interpretation focused on the employment group-by-reserve-building-subscale interaction. To address the third research question, the Series 1 through 4 RE models were computed separately by the employment group using their baseline employment status, and interpretation focused on the main effects of reserve-building activities. For ease of comparison, the same demographic covariates retained for the full sample (i.e., for Research Question 2) were retained for each employment group.

Statistical analyses were implemented using Stata version 17 (StataCorp, 2021), IBM SPSS version 28 (IBM, 2021), and Microsoft Excel.

## Results

### Sample

The study sample included 82% female, 63% married/cohabiting, and 94% white subjects. Over half of the sample had a college or graduate degree, yet 41% reported having some difficulty paying bills. Participants had a mean age of 56 years, had over three comorbidities excluding depression, had an average body mass index of ~30 kg/m^2^, and had been diagnosed with a chronic condition (if applicable) an average of 15 years ago. At baseline, 274 individuals reported being employed; 229 were retired; 83 reported being unemployed; and 175 reported being disabled from working due to a medical condition.

### Employment group differences in reserve-building activities at baseline

**Figure 1:**
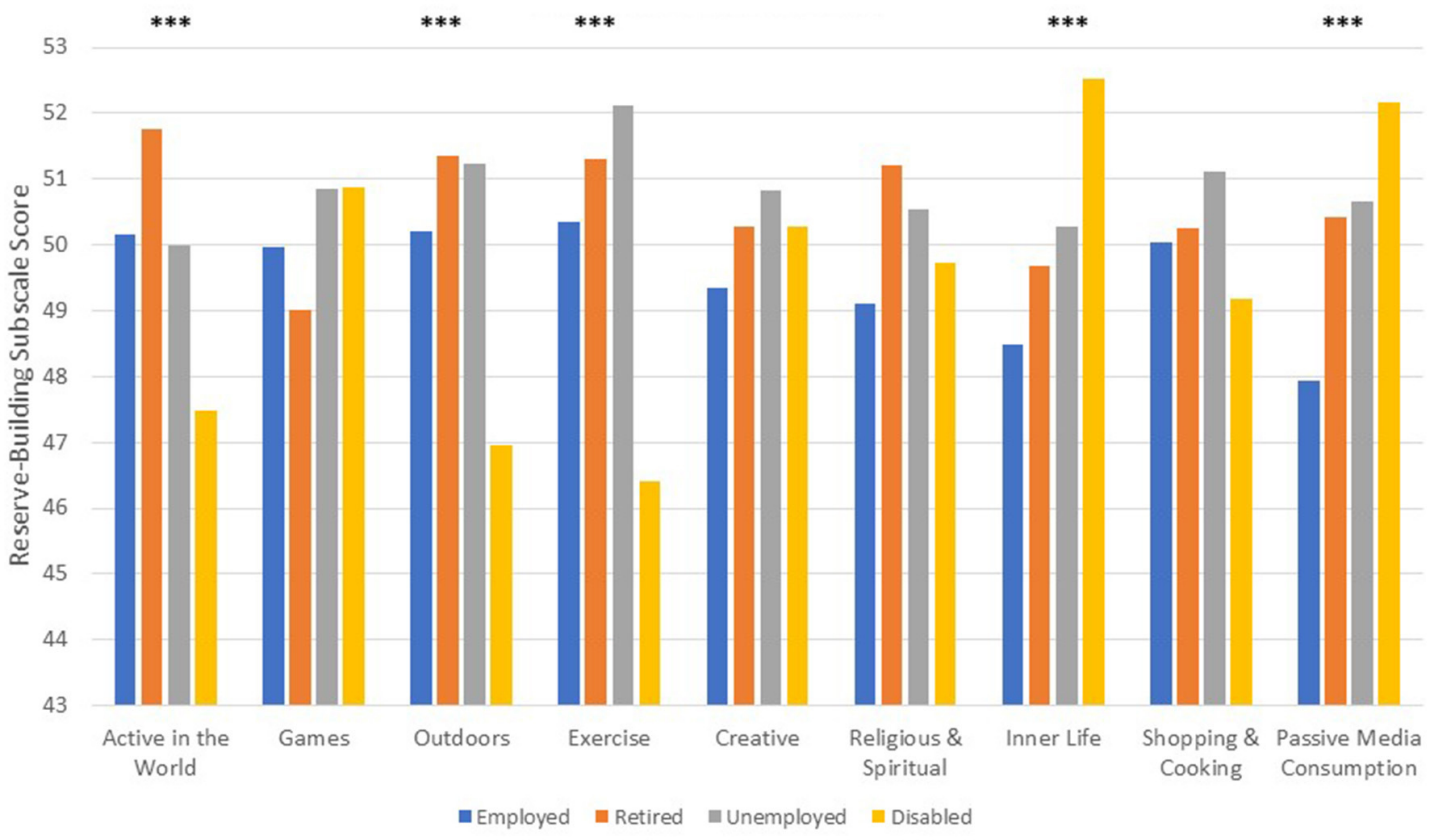
Reserve-building activity means at baseline by employment group. ***p < 0.0001.

Figure 1 displays the unadjusted means for each current reserve-building subscale by employment group. Disabled participants reported substantially lower levels of Active in the World, Outdoors, and Exercise and substantially higher levels of Inner Life and Passive Media Consumption than all other groups (*p* < 0.0001 in all cases).

### Intercorrelations among reserve-building activity subscales

Table 2 provides the intercorrelation matrices for the overall sample and by employment group, with conditional formatting to indicate the effect size of the correlation coefficient using Cohen's cutoffs for small, medium, and large effect sizes (Cohen, 1992). In all cases, multicollinearity was not likely to be a problem in subsequent multivariate models as the intercorrelations were well below the cutoff of 0.80, as recommended by Berry and Feldman (1985). The patterns of intercorrelations were somewhat distinct in the disabled and unemployed groups as compared to the other groups, with more medium effect-size intercorrelations in the former groups. These patterns suggest that, among the disabled and unemployed participants, engaging in any reserve-building activity is associated with more frequent engagement in other reserve-building activities.

Table 2Inter-correlation matrics of reserve-building activities overall and by employment group.

**Table:** 

	**Active in the World**	**Games**	**Outdoors**	**Exercise**	**Creative**	**Religious and Spiritual**	**Inner Life**	**Shopping/ Cooking**	**Passive Media Consumption**
**Overall (*****n*** = **771)**									
Active in the World	1	0.13	0.24	0.23	0.28	0.21	0.04	0.23	−0.01
Games	0.13	1	0.08	0.00	0.26	0.11	0.07	0.13	0.12
Outdoors	0.24	0.08	1	0.43	0.30	0.10	0.05	0.34	−0.12
Excercise	0.23	0.00	0.43	1	0.19	0.10	0.01	0.24	−0.13
Creative	0.28	0.26	0.30	0.19	1	0.25	0.19	0.22	0.00
Religious and Spiritual	0.21	0.11	0.10	0.10	0.25	1	0.07	0.14	0.00
Inner Life	0.04	0.07	0.05	0.01	0.19	0.07	1	0.06	0.14
Shopping and Cooking	0.23	0.13	0.34	0.24	0.22	0.14	0.06	1	0.07
Passive Media Consumption	−0.01	0.12	−0.12	−0.13	0.00	0.00	0.14	0.07	1
**Employed (*****n*** = **274)**									
Active in the World	1	0.20	0.23	0.12	0.32	0.20	0.17	0.19	0.05
Games	0.20	1	0.04	−0.04	0.24	0.15	0.13	0.14	0.22
Outdoors	0.23	0.04	1	0.40	0.29	0.15	0.05	0.28	−0.14
Excercise	0.12	−0.04	0.40	1	0.27	0.14	0.07	0.15	−0.08
Creative	0.32	0.24	0.29	0.27	1	0.34	0.26	0.18	−0.07
Religious and Spiritual	0.20	0.15	0.15	0.14	0.34	1	0.13	0.14	−0.03
Inner Life	0.17	0.13	0.05	0.07	0.26	0.13	1	−0.04	0.14
Shopping and Cooking	0.19	0.14	0.28	0.15	0.18	0.14	−0.04	1	0.12
Passive Media Consumption	0.05	0.22	−0.14	−0.08	−0.07	−0.03	0.14	0.12	1
**Retired (*****n*** = **229)**									
Active in the World	1	0.11	0.18	0.21	0.26	0.18	−0.07	0.18	0.03
Games	0.11	1	0.10	0.02	0.23	−0.02	−0.01	0.04	0.05
Outdoors	0.18	0.10	1	0.42	0.27	−0.03	−0.04	0.42	−0.05
Excercise	0.21	0.02	0.42	1	0.11	0.04	0.00	0.25	−0.18
Creative	0.26	0.23	0.27	0.11	1	0.16	0.10	0.17	0.06
Religious and Spiritual	0.18	−0.02	−0.03	0.04	0.16	1	−0.03	0.00	0.01
Inner Life	−0.07	−0.01	−0.04	0.00	0.10	−0.03	1	0.04	0.11
Shopping and Cooking	0.18	0.04	0.42	0.25	0.17	0.00	0.04	1	−0.02
Passive Media Consumption	0.03	0.05	−0.05	−0.18	0.06	0.01	0.11	−0.02	1
**Unemployed (*****n*** = **83)**									
Active in the World	1	0.24	0.20	0.26	0.26	0.11	0.11	0.28	−0.08
Games	0.24	1	0.35	0.06	0.44	0.24	0.16	0.43	0.09
Outdoors	0.20	0.35	1	0.39	0.35	0.17	0.12	0.39	−0.20
Excercise	0.26	0.06	0.39	1	0.01	−0.01	0.00	0.21	−0.02
Creative	0.26	0.44	0.35	0.01	1	0.26	0.29	0.25	0.02
Religious and Spiritual	0.11	0.24	0.17	−0.01	0.26	1	0.08	0.24	−0.09
Inner Life	0.11	0.16	0.12	0.00	0.29	0.08	1	0.08	0.07
Shopping and Cooking	0.28	0.43	0.39	0.21	0.25	0.24	0.08	1	0.04
Passive Media Consumption	−0.08	0.09	−0.20	−0.02	0.02	−0.09	0.07	0.04	1
**Disabled (*****n*** = **175)**									
Active in the World	1	0.06	0.27	0.34	0.29	0.32	0.02	0.32	−0.08
Games	0.06	1	0.02	0.04	0.23	0.20	0.01	0.07	0.12
Outdoors	0.27	0.02	1	0.41	0.35	0.20	0.22	0.29	−0.08
Excercise	0.34	0.04	0.41	1	0.30	0.17	0.00	0.36	−0.13
Creative	0.29	0.23	0.35	0.30	1	0.23	0.12	0.34	0.02
Religious and Spiritual	0.32	0.20	0.20	0.17	0.23	1	0.12	0.24	0.04
Inner Life	0.02	0.01	0.22	0.00	0.12	0.12	1	0.23	0.10
Shopping and Cooking	0.32	0.07	0.29	0.36	0.34	0.24	0.23	1	0.13
Passive Media Consumption	−0.08	0.12	−0.08	−0.13	0.02	0.04	0.10	0.13	1

**Table:** 

**Effect size**						
Large	Medium	Small		Small	Medium	Large
≤-0.50	−0.49 to −0.30	−0.29 to −0.10	−0.09 to 0.09	0.10 to 0.29	0.30 to 0.49	≥0.50
						

### Reserve-building activities that differentiated the level of baseline depression by employment group

**Figure 2:**
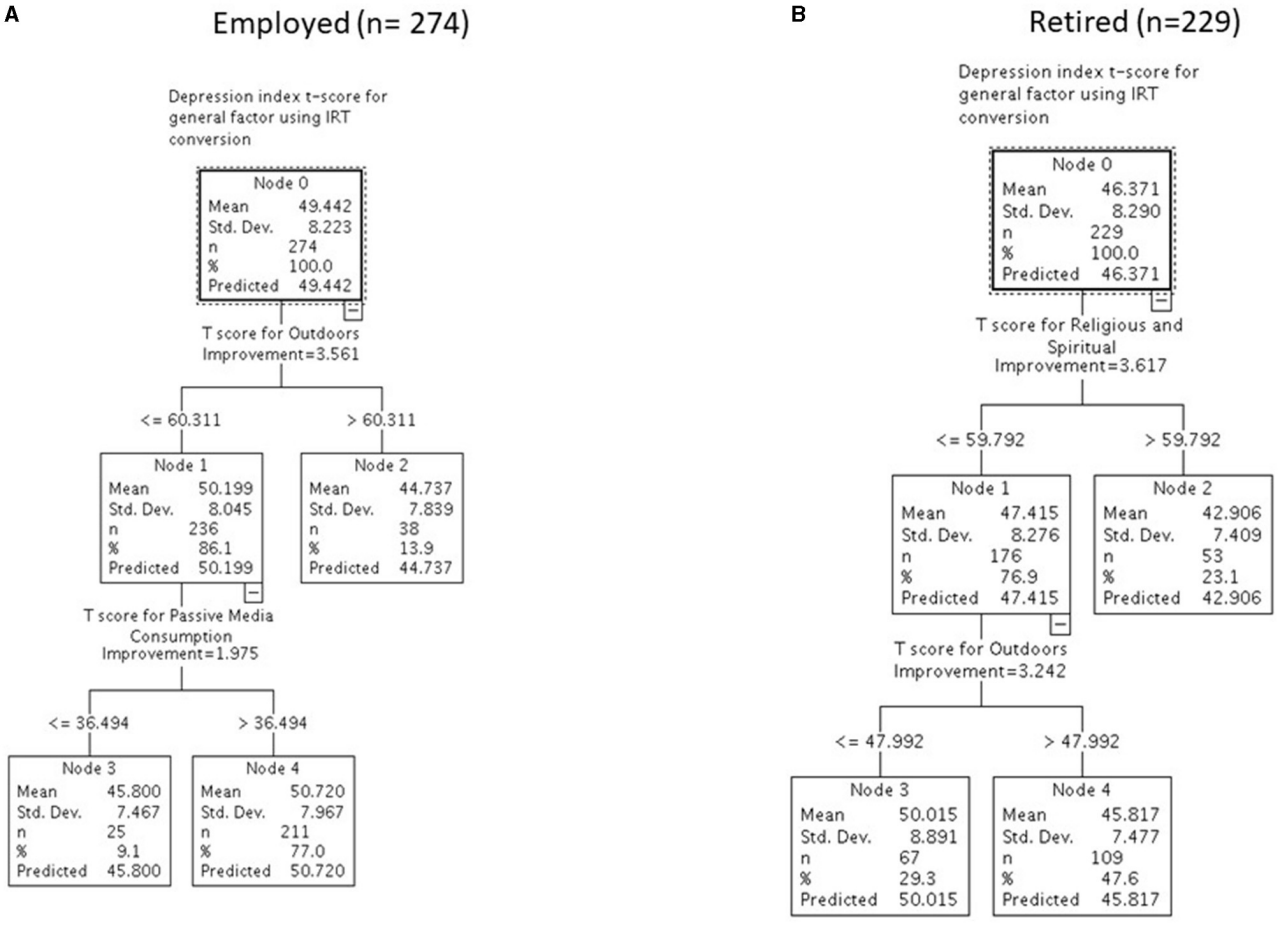
Differentiating levels of baseline depression with reserve-building for employed (*n* = 274) **(A)** and retired (*n* = 229) **(B)** participants.

**Figure 3:**
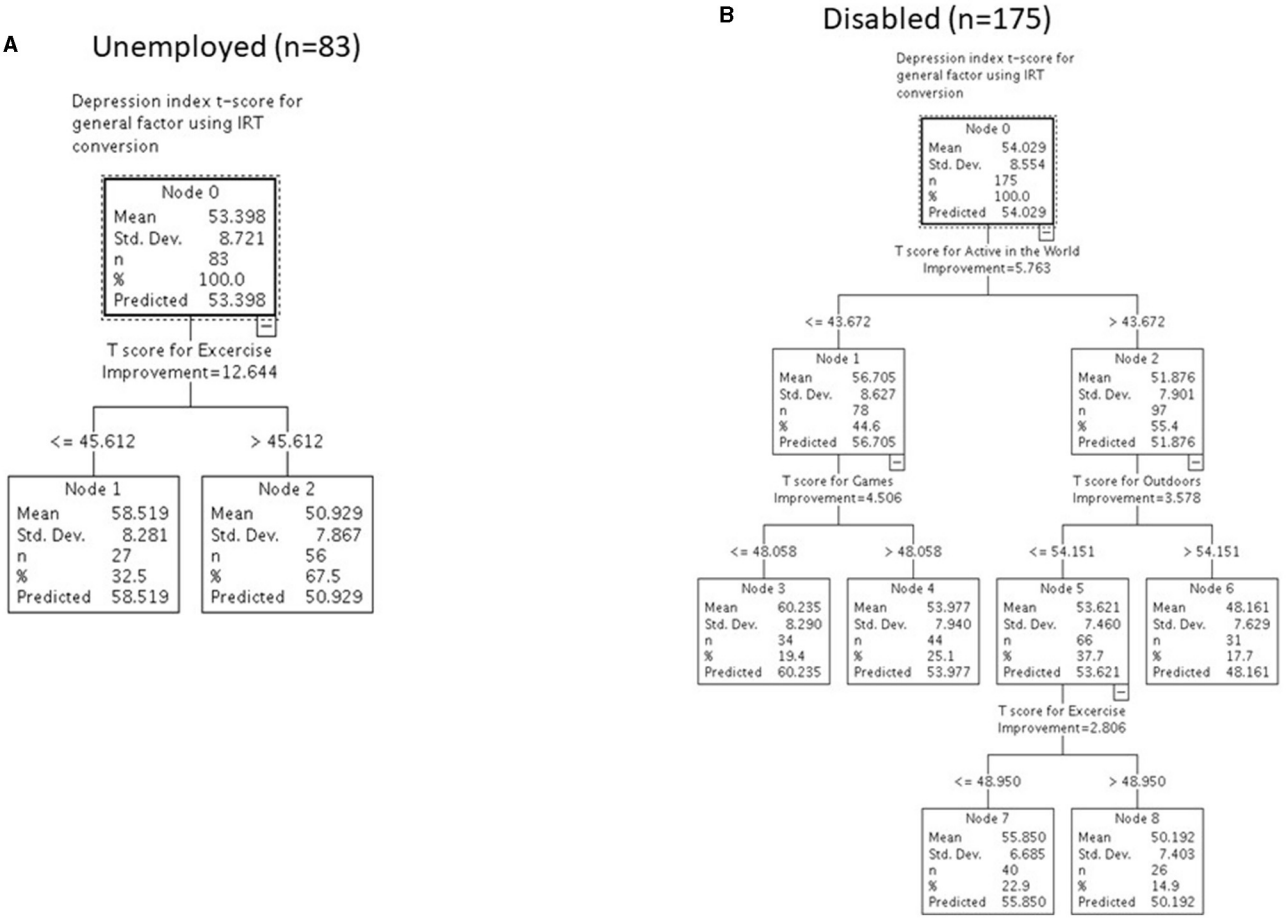
Differentiating levels of baseline depression with reserve-building for unemployed (*n* = 83) **(A)** and disabled (*n* = 175) **(B)** participants.

The CART models for the employed and retired (Figures 2A, B) and unemployed and disabled participants (Figures 3A, B) reveal very different overall group means for baseline depression at Node 0. Retired participants had the lowest reported depression at Node 0, followed by employed, unemployed, and disabled participants (means of 46, 49, 53, and 54). Different reserve-building activities were retained in the four groups' CART models as well. Employed participants reported lower depression if they engaged in more Outdoor reserve-building activities, roughly equivalent to spending time outdoors more than a few times a month; doing outdoor activities with their hands more than two to three times per week; and doing home improvements more than two to three times per week (mean of 45; Figure 2A). Among those with lower engagement in Outdoor activities, lower Passive Media Consumption was associated with lower depression—roughly equivalents to watching movies less than once per week, <1–2 h of television per day, and browsing the internet <1–2 h per day (mean of 46; Figure 2A).

For retired participants, engaging in more Religious/Spiritual activities was associated with the lowest reported depression, roughly associated with engaging in religious and spiritual activities independently at least once a week; group religious or spiritual activities at least once a week; and singing or playing musical instruments at least once a week (mean of 43). Among those less engaged in Religious/Spiritual activities, if they also engaged in Outdoor pursuits (i.e., roughly equivalent to, at a minimum, spending time outdoors about once a month; doing outdoor activities with their hands at least once a week; and doing home improvements at least once a week), their reported depression score was slightly higher (mean of 46) but still below the cut-point for depression. It was substantially worse if they did not engage in Outdoor pursuits (mean of 50).

For unemployed participants, the only reserve-building activity that was retained in the model was Exercise. The overall group had an average depression score of 53. When segmented according to level of Exercise, those who engaged in little exercise had an average depression score of 59 compared to those who engaged in more exercise (i.e., roughly equivalent to reporting, at minimum, engaging in exercise, sports, or dance about two to three times per week and reporting some combination of mild, moderate, and strenuous exercise at least 3 days per week), who had an average depression score of 51.

In contrast, disabled participants' reported depression was associated with more aspects of reserve-building. Engaging more in Active in the World, Games, Outdoor, and Exercise activities was associated with lower depression scores. For this group, the lowest depression scores (mean of 48) were found among those who engaged in Active in the World (i.e., roughly equivalent to going to lectures, concerts, theater, or museums; traveling or going on (day) tours; and doing volunteer work or participating as a volunteer in an organization at least one or more times in the past 6 months) and Outdoor (i.e., roughly equivalent to spending time outdoors more than a few times a month; doing outdoor activities with their hands more than two to three times per week; and/or doing home improvements more than two to three times per week) activities, although engaging in Exercise was associated with a lower depression score than the disabled group's average (i.e., roughly equivalent to reporting, at minimum, engaging in exercise, sports, and dance about two to three times per week and some combination of mild, moderate, and vigorous exercise at least 5 days per week). Engaging more in Games (i.e., at a minimum, playing video games, including games on your phone or computer for 1–2 h per day; doing puzzles or crossword games once per month; and playing card games or board games once a month) was associated with a modest decrease in depression levels for those with low Active in the World scores.

### Buffering contrasts with employed participants in depression trajectories over time

**Figure 4:**
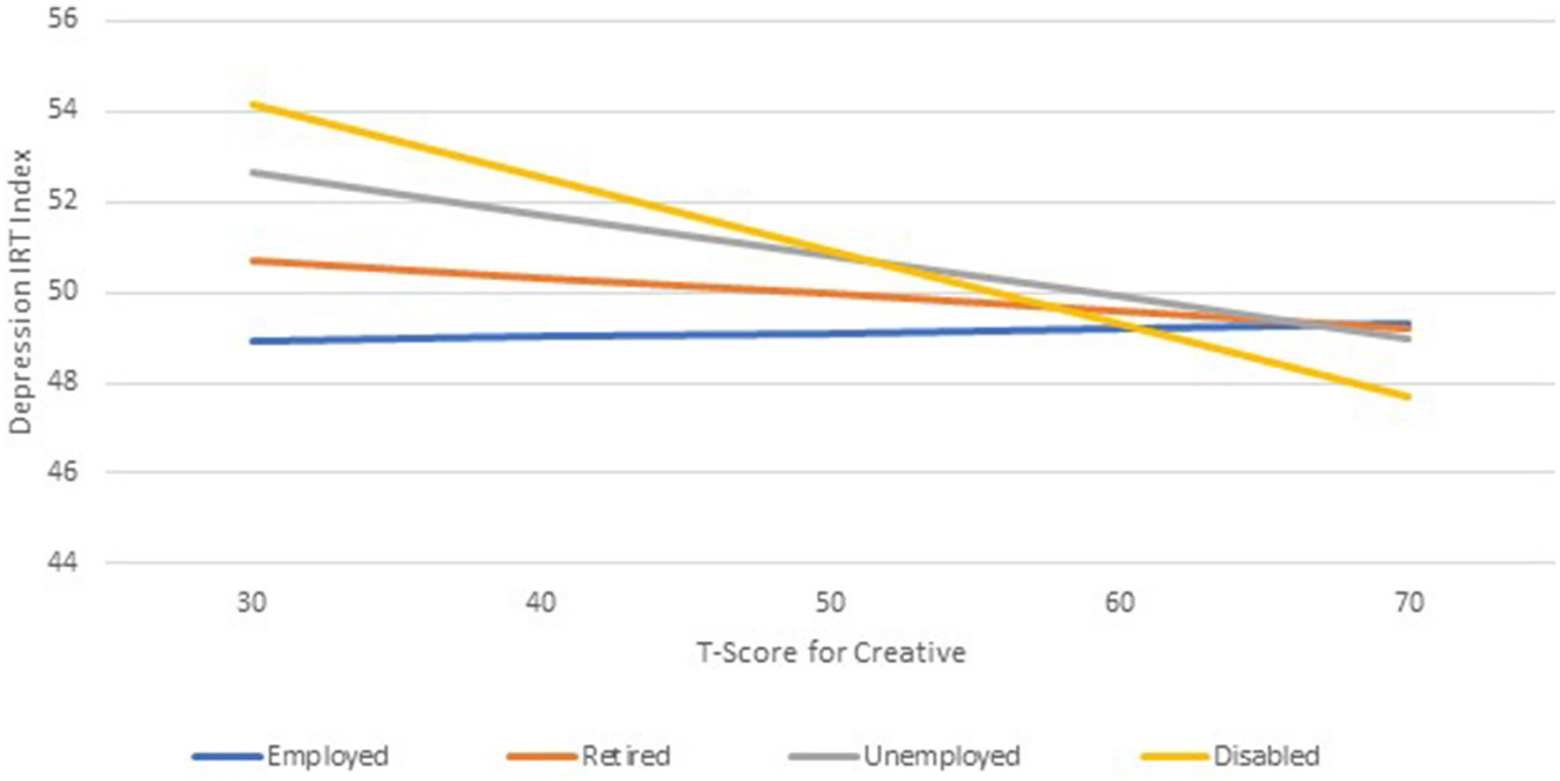
A significant two-way interaction between the employment group and creative reserve-building activities.

The results of the RE models that included the whole sample and focused on the employment group-by-reserve-building-subscale interactions revealed a significant two-way interaction between the disabled group and the creative group (Table 3). Specifically, creative activities provided a substantial buffering effect on depression for disabled participants in comparison to employed participants, after adjusting for covariates. When they did not engage in these pursuits, they reported higher depression of over 0.6 standard deviations than when they engaged regularly in such pursuits (Figure 4). This reflects a medium effect size, which is a clinically important difference (Norman et al., 2003). In the overall sample, reserve-building activities characterized as Outdoor, Exercise, and Religious/Spiritual activities were associated with reduced depression overall (i.e., significant main effects; Table 3).

**Table 3:** Final RE Model predicting depression index with significant reserve-building main effects and interactions after removing those that were no longer significant in earlier model.

**Parameter**	***b***	**SE**	***t***	**Sig**.
Intercept	69.95	2.65	26.39	**0.000**
Time	0.00	0.00	−1.32	0.188
Age (at baseline)	−0.16	0.02	−6.98	**0.000**
Comorbidities (at baseline)^*^	0.30	0.12	2.45	**0.015**
**Difficulty paying bills (ref: not at all) (at baseline)**				
Slightly	2.15	0.66	3.26	**0.001**
Moderately	4.16	0.89	4.66	**0.000**
Very	6.35	1.16	5.50	**0.000**
Extremely	8.74	1.53	5.71	**0.000**
**Employment status (ref: employed)**				
Unemployed	6.70	2.80	2.40	**0.017**
Retired	3.16	2.55	1.24	0.217
Disabled	10.33	2.75	3.76	**0.000**
COVID infection status (at baseline)	3.60	1.12	3.21	**0.001**
**Reserve-building at baseline**				
Outdoors	−0.08	0.03	−2.81	**0.005**
Exercise	−0.10	0.03	−3.42	**0.001**
Creative	0.01	0.04	0.23	0.819
Religious/spiritual	−0.12	0.03	−4.41	**0.000**
**Employment group by creative interactions**				
Unemployed-by-creative	−0.10	0.05	−1.84	0.066
Retired-by-creative	−0.05	0.05	−0.93	0.351
Disabled-by-creative	−0.17	0.05	−3.19	**0.001**
**Variance explained**				
Explained variance across all predictors (except time)	*Estimate*			
Proportion of within-person variance explained	0.01			
Proportion of between-person variance explained	0.34			
Total explained variance	0.27			
**Explained variance due to reserve-building only**				
Proportion of within-person variance explained	0.00			
Proportion of between-person variance explained	0.08			
Total explained variance	0.06			

^*^Excluding depression.

Bolded values highlight significant *p*-values.

Among the model covariates, older age was associated with better depression trajectories, whereas the number of comorbidities, difficulty paying bills, and having had COVID-19 at baseline were associated with worse depression trajectories. The final RE model, including reserve-building activities, explained 27% of the variance in depression trajectories above and beyond the null model that included only time, although 6% of the variance was specifically explained by reserve-building activities (Table 3).

### Buffering patterns within employment groups in depression trajectories over time

The results of the RE models computed separately for each employment group revealed that more reserve-building activities played a prominent role in buffering depression for disabled participants as compared to the retired and unemployed groups, and no such role for employed participants (Table 4). For disabled participants, engaging in Religious/Spiritual activities, Outdoor activities, and Game-playing was associated with a significant reduction in depression (*p* < 0.01, 0.05, and 0.05, respectively; Table 4). For retired participants, Religious/Spiritual and Shopping/Cooking activities as hobbies were associated with reduced depression over time (*p* < 0.05 in both cases; Table 4), whereas for unemployed participants, only Exercise buffered depression (*p* < 0.01; Table 4). No reserve-building activity was associated with the depression trajectory for the employed participants.

**Table 4:** Final models predicting depression index by employment group.

**Parameter**	**Employed**		**Unemployed**		**Retired**		**Disabled**	
	***t***	**Sig**.	***t***	**Sig**.	***t***	**Sig**.	***t***	**Sig**.
Intercept	12.50	**0.000**	7.86	**0.000**	9.94	**0.000**	12.29	**0.000**
Time	−1.19	0.233	−1.42	0.156	0.65	0.518	−0.92	0.359
Age (at baseline)	−4.53	**0.000**	−1.36	0.179	−2.44	**0.016**	−3.85	**0.000**
Comorbidities (at baseline)^*^	1.52	0.130	0.16	0.873	2.26	**0.025**	0.63	0.529
**Difficulty paying bills (ref: not at all) (at baseline)**								
Slightly	1.83	0.069	0.04	0.971	1.14	0.255	2.25	**0.026**
Moderately	3.07	**0.002**	1.48	0.144	0.40	0.692	2.22	**0.028**
Very	3.10	**0.002**	0.70	0.489	1.86	0.065	4.30	**0.000**
Extremely	2.56	**0.011**	4.22	**0.000**			2.70	**0.008**
COVID infection status (at baseline)	−0.59	0.558	1.83	0.073	3.59	**0.000**	1.64	0.103
**Reserve-building at baseline**								
Active in the World	0.80	0.422	−1.92	0.060	−1.84	0.067	−0.50	0.618
Games	0.42	0.676	−0.39	0.694	−0.80	0.425	−2.04	**0.044**
Outdoors	−0.92	0.357	−0.01	0.990	−0.36	0.716	−2.35	**0.020**
Exercise	−1.05	0.295	−2.84	**0.006**	−1.20	0.230	−0.92	0.359
Creative	−1.34	0.181	−1.97	0.053	−0.98	0.328	0.82	0.412
Religious/spiritual	−1.62	0.107	−0.10	0.918	−2.42	**0.017**	−2.93	**0.004**
Inner Life	1.02	0.308	0.24	0.808	0.25	0.799	1.25	0.214
Shopping/Cooking	−0.97	0.332	1.82	0.073	−2.01	**0.046**	−0.96	0.337
Passive Media Consumption	−0.37	0.713	0.75	0.457	0.37	0.709	0.70	0.486
**Variance explained**								
Explained variance across all predictors (except time)	*Estimate*		*Estimate*		*Estimate*		*Estimate*	
Proportion of within-person variance explained^†^	0.00		0.00		0.00		0.00	
Proportion of between-person variance explained	0.20		0.47		0.21		0.36	
Total explained variance	0.15		0.36		0.17		0.27	
**Explained variance due to reserve-building only**								
Proportion of within-person variance explained^†^	0.00		0.00		0.00		0.00	
Proportion of between-person variance explained	0.02		0.15		0.09		0.15	
Total explained variance	0.02		0.11		0.07		0.11	

SE, standard error.

^*^Excluding depression.

^†^Within-person variance should be zero because all predictors are time-invariant (i.e., collected only at baseline). Bolded values highlight significant *p*-values.

Among the model covariates, older age was associated with less depression for all but unemployed participants, whereas the number of comorbidities and having had COVID-19 at baseline were associated with worse depression only among retired participants. Difficulty paying bills was associated with worse depression for all but retired participants. Overall, the RE models explained the most variance among unemployed participants, followed by disabled participants, retired participants, and finally employed participants (total explained variance above and beyond the null model that included only time = 36%, 27%, 17%, and 15%, respectively). Reserve-building activities specifically explained 11% of the variance in depression for disabled and unemployed participants, 7% for retired participants, and 2% for employed participants (Table 4).

## Discussion

**Figure 5:**
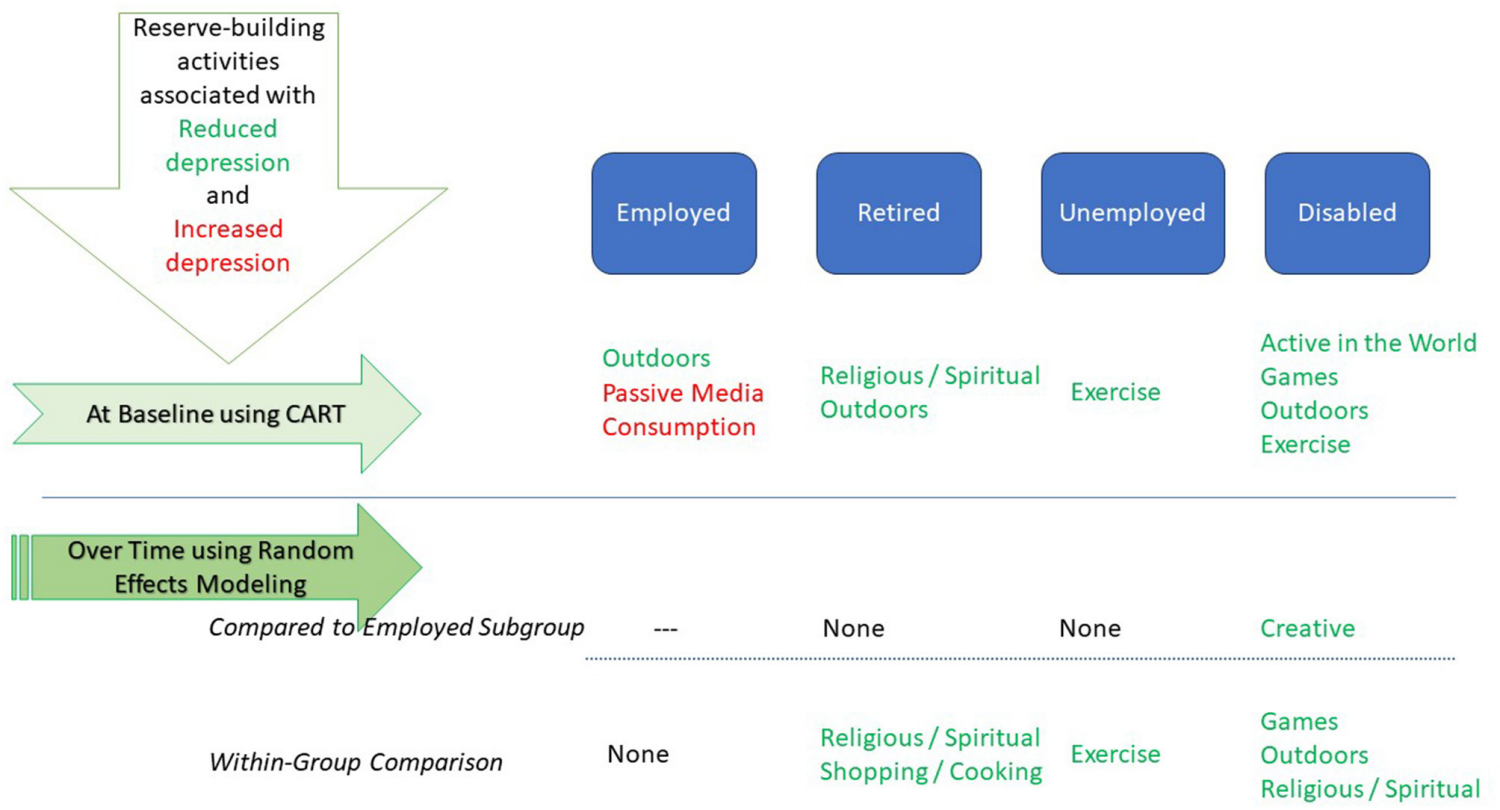
Summary of study findings.

To the best of our knowledge, this is the first study to address the relationship between employment status, reserve-building activities, and mental health during the pandemic. In this study of depression trajectories during the COVID-19 pandemic, remaining employed was found to be a highly protective factor against depression. Figure 5 provides a visual summary of the study's major findings. While engaging in Outdoor activities, Exercise, and Religious/Spiritual activities was associated with reduced depression over time in the overall sample, there were notable employment-group differences in the variety and nature of reserve-building activities that appeared to buffer reported depression during the pandemic. Of note, people who were disabled from work due to a medical condition differed from the other employment groups in each of the analyses. While they endorsed lower levels of Active in the World, Outdoors and Exercise and higher levels of Inner Life and Passive Media Consumption than the other employment groups, their engagement in the former three activities and Games was revealed to distinguish depression subgroups within this employment group.

Among the disabled group, engaging in any reserve-building activities was also associated with engaging in more such activities, which was similar to the unemployed participants but distinct from the retired and employed participants. When compared to the employed group, only the disabled group's level of depression was found to be buffered by engaging in Creative activities, despite no substantial group differences in engaging in such activities. Furthermore, the disabled group's engagement in Religious/Spiritual, Outdoors, and Games was associated with substantially reduced depression, which was different from the other employment groups. These findings highlight the importance of engaging in whatever reserve-building activities are accessible and pleasurable as a way to mitigate depression over time. Their importance was particularly salient for those people who are disabled from work and suffer from stable depression more than those in other employment groups.

In contrast, reserve-building activities were not implicated at all as buffers for employed participants in the longitudinal model. It is possible that, particularly during the pandemic, employed participants had much less discretionary time due to the many aspects of life activities that were made more complicated and time-consuming than normal times. For example, grocery shopping early in the pandemic took longer due to the perceived need to wash everything in case the virus was present on the packaging, clothing worn in the store, etc. Because schools were closed during the early part of the pandemic, home-schooling children may have been required, further reducing the discretionary time of employed individuals. However, at baseline, Outdoor activities and Passive Media Consumption were associated with reduced and higher levels of depression, respectively, for the employed group. Spending time outdoors and consuming media were common ways to spend time during the early months of the pandemic (i.e., at baseline), as Outdoor and solitary activities were considered low-risk activities for contracting COVID-19.

Overall, employed individuals reported the lowest levels of depression in the sample. This finding supports the collateral benefits of employment in the present sample. The benefits of employment may not have emerged in the same way in a sample of lower socioeconomic status, particularly during COVID-19, where such people might be unable to work from home or have less control over their working conditions. For example, remaining employed provided usual access to work-related social connections, a welcome distraction from the pandemic, and ensured cognitive stimulation in ways other than reserve-building activities.

These findings highlight the importance of targeted interventions to help people who are disabled from work retain reserve-building activities despite limited financial resources. In work done on people with multiple sclerosis, scores reflecting current reserve-building activities had a similar range across disability groups but were comprised of different specific activities, indicating that patients maintained their reserve with different activities as the disease progressed (Schwartz et al., 2013a). Coaching interventions might help individuals who are disabled from work identify activities that are both stimulating and of interest to them, and then find ways of engaging in such activities that are within their financial means. For example, research has documented that being Active in the World, Outdoor activities, and Exercise are strong predictors of resilience (Schwartz et al., 2019), which is similar to the current study's findings. These types of activities can be done with little or no cost, but individuals may need to be made aware of the benefits of such activities and assisted in learning about such low-cost or free activities in their community. Future research is needed to explore the long-term effects of reserve-building activities on mental health and to identify effective intervention strategies for individuals with limited financial resources.

## Limitations

This study provided a comprehensive investigation of how engaging in reserve-building activities may buffer depression during the COVID-19 pandemic as a function of employment group. Despite its substantial sample with longitudinal data, several caveats should be noted. First, reserve-building data were available only at baseline in the present analysis, thereby limiting our understanding of how these activities might fluctuate over time with depression. Future research is needed to examine the benefits of reserve-building activities that people adopted to cope with the pandemic. Second, given the quasi-experimental nature of the study (i.e., the pandemic context and person-level characteristics were not randomly assigned), we cannot make strict causal inferences from the study findings. Study findings should thus be interpreted with caution. Additional research using a clinical trial design would be useful for establishing causal relationships between reserve-building and depression in the context of a coaching intervention, for example; although the pandemic context and person-level characteristics would still not be able to be randomly assigned. We are also unable to comment knowledgeably on the reasons underlying employment-group differences on the link between reserve-building activities and depression. Future targeted research, possibly utilizing qualitative methods, might investigate the reasons underlying employment-group differences as well as what factors contribute to an individual choosing one type of reserve-building activity over another. Furthermore, the sample sizes of the employment groups varied, with the smallest number reporting being unemployed (*n* = 83). It is possible that one or more of the analyses may have been affected by these sample size variations. For example, the CART models yielded fewer branches in the unemployed subgroup vs. the other employment groups. Accordingly, the study results should again be interpreted with caution. Finally, we are aware that working during COVID-19 was a major stressor for a large segment of the population. Our study sample likely represents those individuals who participated (e.g., predominantly white, married, college-educated, and having relatively low difficulty paying bills). Its generalizability may be limited, so replication is warranted to determine whether the study findings apply to different demographic groups with varying socioeconomic backgrounds.

## Conclusion

This study revealed a beneficial effect of reserve-building activities in buffering depression over time during the COVID-19 pandemic, particularly for people who were disabled from employment due to a medical condition. It documented that even if such individuals engaged in lesser amounts of such activities as compared to other employment groups, the buffering effect was substantial. Given the low-cost and accessible nature of reserve-building activities, it would be worthwhile to encourage such activities for disabled individuals.

## Data availability statement

The datasets presented in this article are not readily available because the study data are confidential and thus not able to be shared. Requests to access the datasets should be directed at: carolyn.schwartz@deltaquest.org.

## Ethics statement

The studies involving humans were approved by New England Independent Review Board. The studies were conducted in accordance with the local legislation and institutional requirements. The participants provided their written informed consent to participate in this study.

## Author contributions

CS: Conceptualization, Data curation, Formal analysis, Methodology, Project administration, Resources, Supervision, Validation, Visualization, Writing—original draft, Writing—review & editing. KB: Conceptualization, Data curation, Formal analysis, Methodology, Writing—original draft, Writing—review & editing. BR: Conceptualization, Validation, Writing—review & editing.
